# Trends in early-onset cancer incidence rates in the Nordic countries (2003–2022): a population-based study

**DOI:** 10.1016/j.lanepe.2026.101858

**Published:** 2026-09-12

**Authors:** Anna LV. Johansson, Simon M. Kønig, Rafael Cardoso, Henrik Møller, Giske Ursin, Anna Skog, Åsa Persson, Helgi Birgisson, Lára Kristjánsdóttir, Tomas Tanskanen, Elna D. Dahl-Olsen, Freddie Bray, Søren Friis, Mats Lambe

**Affiliations:** a Department of Medical Epidemiology and Biostatistics, Karolinska Institutet, Stockholm, Sweden; b Cancer Registry of Norway, Norwegian Institute of Public Health, Oslo, Norway; c Danish Cancer Institute, Danish Cancer Society, Copenhagen, Denmark; d The Danish Healthcare Quality Institute, Aarhus, Denmark; e Danish Center for Health Services Research, Aalborg University, Aalborg, Denmark; f Swedish Cancer Register, National Board of Health and Welfare, Stockholm, Sweden; g Icelandic Cancer Registry, Reykjavík, Iceland; h Finnish Cancer Registry, Cancer Society of Finland, Helsinki, Finland; i Faroese Cancer Registry, Faroese Health Authority, Torshavn, Faroe Islands; j Cancer Surveillance Branch, International Agency for Research on Cancer, Lyon, France

**Keywords:** Cancer, Incidence, Trend, Early-onset, Age, NORDCAN, Nordic countries

## Abstract

**Background:**

Increasing incidence rates of early-onset cancer at ages 20–49 years have been reported in several countries, especially for colorectal, kidney and breast cancer. We examined recent incidence trends of early-onset cancer in Denmark, Finland, Iceland, Norway, Sweden, Greenland, and the Faroe Islands across 18 major cancer sites and compared to trends at ages 50–84 years.

**Methods:**

We utilised the NORDCAN database comprising incident cancer cases among individuals aged 20–84 years during 2003–2022. Age-standardised (Nordic population 2000) incidence rates (ASR) per 100,000 person-years were calculated and linear regression applied to the log of ASRs to quantify the estimated annual percent change (EAPC) by age during the 20-year period.

**Findings:**

Early-onset cancer ASRs increased significantly for 12 of 18 evaluated cancer sites. The largest increases were observed for colon, kidney, and thyroid cancer (EAPCs > 2%), with the highest EAPC observed for colon cancer at <35 years. Increases were also observed for melanoma, multiple myeloma, rectal, prostate, and liver cancer (EAPCs 1–2%). For thyroid cancer, the increases were similar among individuals aged 20–49 and 50–84 years (3.8% vs 4.0%), while for colon and kidney cancer, the increases were higher among individuals aged 20–49 years (colon: 2.7% vs 0.4%; kidney: 2.7% vs 1.2%). In contrast, the increases in melanoma and liver cancer were even greater among those above 50 (melanoma: 2.0% vs 4.2%; liver: 1.5% vs 2.6%). For cancers of the rectum, prostate, corpus uteri, and cervix uteri, the increases in early-onset incidence rates were contrasted by decreasing trends among older individuals. The relative increases translated into 9174 additional early-onset cases (from 47,491 in 2003–2007 to 56,665 in 2018–2022).

**Interpretation:**

We found evidence of increasing incidence rates of early-onset cancer across 18 major cancer sites in the Nordic countries, especially for colon, kidney, and thyroid cancer. Explanations are likely multi-fold and cancer-specific, including changes in early-in-life risk factor exposures in recent generations and increased diagnostic intensity, in particular for thyroid cancer.

**Funding:**

Swedish Research Council and Swedish Cancer Society.

**Research in context**

**Evidence before this study**

We searched PubMed from database inception to March, 2026, for articles published in English on incidence trends of early-onset cancer in comparison to older age groups using the search terms (“early-onset cancer” OR “early-onset malignancy”) AND (“incidence” OR “trend”). Reports on a rise in incidence of early-onset cancer, defined as cancer in adults below the age of 50 years, have been published from several countries. No comprehensive assessment of early-onset cancer trends in the Nordic countries has been reported.

**Added value of this study**

In a population-based setting including seven Nordic countries (Denmark, Finland, Iceland, Norway, Sweden, the Faroe Islands, and Greenland), we utilised harmonised cancer registry data from the NORDCAN database covering the study period 2003–2022 with comparison to historic trends from 1978 onwards. We found that early-onset cancer incidence had increased during 2003–2022 for 12 of 18 common cancers, with the largest increases observed for thyroid, colon, and kidney cancers. Contrasting trends, with increasing early-onset vs decreasing late-onset cancer, were observed for cancers of the rectum, prostate, corpus uteri, and cervix uteri. The age-specific gradient in early-onset incidence varied across cancer sites with the largest increases in ages <35 years for colon and breast cancers, and in ages 35–49 years for melanoma, thyroid, and prostate cancer. Furthermore, the rising trends were mainly observed in birth cohorts from the 1980s onwards, however, for colon, melanoma, pancreas, thyroid, and breast cancers, the increases had already started in younger birth cohorts.

**Implications of all the available evidence**

The findings indicate a rising burden of early-onset cancer in the Nordic countries with substantial variation across 18 major cancer sites. Although absolute incidence rates are low in comparison to older individuals, the findings highlight the need for further research to identify modifiable early-in-life risk factors associated with early-onset cancer and to inform primary prevention strategies among younger populations.

## Introduction

Increasing cancer incidence rates among young individuals aged 20–49 years, so-called early-onset cancer, have been occurring in several countries since the 1990s.^1^, ^2^, ^3^, ^4^, ^5^, ^6^ Of special concern are cancer sites where the increasing rates occur either exclusively in this younger age group or are more pronounced than corresponding trends at older ages.

While the changing scale and profile of cancer among younger individuals has received relatively limited attention up until recently, the observed rises in early-onset cancer have potentially profound implications on societal and healthcare costs. Young individuals have many healthy life-years to lose, and a cancer diagnosis may lead to life-long cancer-related health problems, as well as productivity losses due to premature morbidity and mortality. In addition, the higher risk of cancer in younger generations may be carried forward, leading to future increased burden of disease and costs also among the older populations.

The mechanisms underlying these trends are likely to be multifactorial and vary by cancer type.^7^ Recent studies, particularly in the U.S., have suggested that the increasing incidence trends of early-onset cancers were particularly pronounced for obesity-related cancers, thereby suggesting that obesity could be an explanation of the trends.^2^^,^^4^^,^^5^ Additional risk factors include sedentary lifestyle (physical inactivity), changes in dietary habits, smoking and alcohol consumption, changing reproductive patterns, ultraviolet (UV) radiation, and genetic predisposition.^1^^,^^7^ Importantly, the recent upward trends may partially reflect more frequent use of increasingly sensitive diagnostic methods.^8^^,^^9^

In international comparisons, the Nordic countries represent a region characterised by high cancer incidence yet comparatively low mortality rates, a pattern observed in both younger and older age groups.^10^^,^^11^ The Nordic countries are culturally comparable, high-income societies with universal tax-funded healthcare systems, national cancer strategies, standardised cancer care pathways, and national screening programmes.^12^ While risk factor patterns are broadly similar across the Nordic countries, changes in lifestyle-related determinants in younger generations^13^^,^^14^ underscores the importance of evaluating incidence trends of early-onset cancer. To our knowledge, no previous comprehensive assessment of early-onset cancer trends has been reported from the Nordic region, and studies from other settings have generally reported trends up to 2019. Such findings are key to inform health policy, preventive efforts and clinical practices across the region, while also providing an important benchmark for international comparisons.

Using population-based cancer registry data from Denmark, Finland, Iceland, Norway, Sweden, the Faroe Islands, and Greenland obtained from the NORDCAN database,^15^ we describe incidence trends of early-onset cancer across 18 major cancer sites in women and men diagnosed at ages 20–49 years between 2003 and 2022, and compare these trends with corresponding incidence patterns in older adults aged 50–84 years. We also investigate age-specific gradients within early-onset cancer using 5-year age groups. Furthermore, we compare recent age-specific incidence trends to historical trends from 1978 onwards to explore potential birth cohort effects.

## Methods

In this population-based study, we used publicly available population-based data from the NORDCAN database, which holds information on cancer cases reported to the national population-based cancer registries in Denmark, Finland, Iceland, Norway, Sweden, the Faroe Islands, and Greenland.^15^^,^^16^ The Nordic cancer registries have a long history of recording cancer in their populations. To capture the recent incidence trends, we included cancers diagnosed in women and men aged 20–84 years from 2003 until 2022, and for comparison to historical trends we also included data from 1978 until 2002. Reporting to the Nordic cancer registries is mandated by law yielding data of high quality and completeness.^17^ In 2018–2022, 92–97% of cancer cases were histologically verified, except for Greenland with 89%.^15^ To ensure comparability across countries and over time, all national cancer registry data included in NORDCAN are processed and harmonised in a federated analysis framework developed by the NORDCAN project group with representatives from each of the Nordic Cancer Registries and the International Agency for Research on Cancer (IARC). In each new version of the NORDCAN database, the IARC multiple primary rules are applied uniformly to each national dataset for both recent and historical years before being combined into NORDCAN.^15^^,^^16^^,^^18^

We identified the 20 most common cancer sites according to crude incidence rates among individuals aged 20–84 years in the Nordic countries during 2003–2022 to reflect the cancer burden among the adult population. The majority of these (15 out of 20) were also among the 20 most common in individuals aged 20–49 years. The cancer sites were defined by the tenth revision of the International Classification of Diseases (ICD-10): lip, oral cavity, pharynx (C00–C14, excluding C10.1), stomach (C16), colon (C18), rectum (C19–C20), liver (C22), pancreas (C25), lung (C33–C34), female breast (C50), corpus uteri (C54), ovary and tubes (C56, C57.0–C57.4), prostate (C61), kidney (C64), bladder and urinary tract (C65–C68, D09.0–D09.1, D30.1–D30.9, D41.1–D41.9), melanoma of skin (C43), non-melanoma of the skin (C44), brain and central nervous system (CNS) (C70–C72, D32–D33, D42–D43), thyroid (C73), non-Hodgkin lymphoma (C82–C86, C96), multiple myelomas (C90), and leukaemia (C91–C93, C94.0, C94.2–C94.4, C94.7, C95). Due to known country differences over time in the coding and registration of cancers of the brain and bladder (e.g., non-invasive, benign or unspecified tumours) as well as non-melanoma skin cancer (e.g., basal cell carcinoma and keratoacanthoma), we excluded these three sites. Cancer of the cervix uteri (C53), a malignancy affecting young women and targeted by a screening programme in all Nordic countries, was additionally included.

For the 18 cancer sites included, we extracted counts of cancer cases by age at diagnosis (5-year age groups), year of diagnosis, sex, country, and cancer site from NORDCAN.^15^ Population counts for the at-risk population in each country were obtained as mid-year population counts per year, sex, and age group. Birth cohort was calculated from age and period of diagnosis as cohort = period–age. Due to 1-year period and 5-year age intervals, the birth cohort intervals were slightly misclassified at the boundaries.

### Statistical methods

Crude incidence rates of cancer were estimated as number of cancer cases divided by population counts by age, sex, and year and expressed per 100,000. Age-standardised incidence rates (ASRs) were standardised according to the Nordic standard population in year 2000 as defined by NORDCAN (Supplemental Table S1). In NORDCAN online, additional age standards (World ASR and two European age standards) are also available.

To describe the change in incidence trends, we calculated the estimated annual percent change (EAPC), which is the average annual change of the rate over a given period. EAPC was estimated by fitting a linear regression model of the log rate with year as covariate, which assumes a constant change of the rate during the assessed time period.^19^ The 95% confidence intervals (CI) for EAPC were based on the normal distribution. Firstly, we estimated the EAPC for truncated ASRs at ages 20–49 and 50–84 years over the period 2003–2022 for all 18 cancer sites. Secondly, we estimated the EAPC based on crude incidence rates within 5-year age groups over the period 2003–2022, and separately for 2003–2012 and 2013–2022 for all 18 cancer sites to assess the assumption of log-linear trends. Thirdly, we estimated the EAPC based on ASRs for ages 20–49 and 50–84 per country to assess country-specific differences for the nine cancer sites in which the EAPC at ages <50 years was significantly greater than zero in at least one age group. In the country-specific analyses, the log-linear models were based on available data without any weighting due to low rates, and the uncertainty is reflected in the confidence intervals. Lastly, for those nine cancer sites, we estimated the age-specific incidence rates by period (age-period) and by birth cohort (age-cohort) for the longer period 1978–2022. Results were considered statistically significant when the 95% CI did not include the null value.

The statistical analyses were performed in R software version 4.3.2. The study was approved by the Swedish Ethical Review Authority (DNR 2017/641-31/1, 2024-07257-02).

### Role of the funding source

The funding sources had no role in the study design, data collection, analysis, interpretation of the data, writing of the report, and the decision to submit the paper for publication.

## Results

In total, 2,209,703 incident cancer cases (women: N = 1,065,660; men: N = 1,144,043) were identified across the 18 selected cancer sites in the Nordic countries between 2003 and 2022 (Table 1, Supplemental Table S2). The four largest countries (Denmark, Finland, Norway and Sweden) contributed 98.3% of the early-onset cancer cases and 98.7% of cases at ages 50–84 years. The most common cancer sites were prostate, breast, lung, colon, and melanoma.

**Table 1:** Number of cancer cases by country across 18 cancer sites for men and women combined at ages 20–49 and 50–84 years in 2003–2022.

Cancer sites	Denmark		Finland		Norway		Sweden		Faroe Islands		Greenland		Iceland		Nordic countries	
	20–49 N (%)	50–84 N (%)	20–49 N (%)	50–84 N (%)	20–49 N (%)	50–84 N (%)	20–49 N (%)	50–84 N (%)	20–49 N (%)	50–84 N (%)	20–49 N (%)	50–84 N (%)	20–49 N (%)	50–84 N (%)	20–49 N (%)	50–84 N (%)
Lip, oral cavity and pharynx	1731 (3.3)	15,898 (3.3)	1259 (3.2)	10,125 (2.5)	1089 (2.4)	8971 (2.4)	2002 (2.7)	17,525 (2.5)	13 (3.7)	80 (2.6)	46 (8.3)	210 (7.5)	72 (2.6)	400 (2.1)	6212 (2.9)	53,209 (2.7)
Stomach	809 (1.5)	10,433 (2.2)	792 (2.0)	10,687 (2.6)	611 (1.4)	7635 (2.0)	1071 (1.5)	14,331 (2.0)	8 (2.2)	80 (2.6)	28 (5.1)	149 (5.3)	40 (1.5)	432 (2.3)	3359 (1.6)	43,747 (2.2)
Colon	2542 (4.8)	50,548 (10.6)	2224 (5.7)	31,179 (7.6)	2898 (6.5)	43,631 (11.5)	4003 (5.4)	68,361 (9.7)	22 (6.2)	380 (12.2)	30 (5.4)	420 (15.1)	167 (6.1)	1886 (9.9)	11,886 (5.6)	196,405 (9.8)
Rectum	1562 (3.0)	25,736 (5.4)	1195 (3.0)	19,296 (4.7)	1752 (3.9)	21,379 (5.6)	2267 (3.1)	34,441 (4.9)	18 (5.1)	228 (7.3)	35 (6.3)	217 (7.8)	88 (3.2)	668 (3.5)	6917 (3.2)	101,965 (5.1)
Liver	352 (0.7)	7173 (1.5)	296 (0.8)	8434 (2.1)	329 (0.7)	3991 (1.1)	635 (0.9)	11,751 (1.7)	<5 (NA)	48 (1.5)	7 (1.3)	57 (2.0)	21 (0.8)	316 (1.7)	1642 (0.8)	31,770 (1.6)
Pancreas	769 (1.5)	17,106 (3.6)	698 (1.8)	19,454 (4.8)	620 (1.4)	12,345 (3.3)	946 (1.3)	22,273 (3.2)	5 (1.4)	148 (4.7)	21 (3.8)	143 (5.1)	32 (1.2)	651 (3.4)	3091 (1.4)	72,120 (3.6)
Lung	2598 (4.9)	83,560 (17.5)	1162 (3.0)	46,954 (11.5)	1673 (3.8)	52,397 (13.8)	2063 (2.8)	72,740 (10.3)	8 (2.2)	389 (12.5)	44 (8.0)	846 (30.4)	117 (4.3)	3102 (16.2)	7665 (3.6)	259,988 (13.0)
Breast	15,267 (28.9)	72,471 (15.2)	13,371 (34.1)	71,232 (17.4)	12,894 (28.9)	46,802 (12.3)	24,696 (33.5)	105,209 (14.9)	86 (24.2)	431 (13.8)	139 (25.1)	230 (8.3)	882 (32.3)	3137 (16.4)	67,335 (31.5)	299,512 (15.0)
Cervix uteri	3907 (7.4)	3143 (0.7)	1660 (4.2)	1484 (0.4)	3714 (8.3)	2619 (0.7)	5228 (7.1)	4385 (0.6)	37 (10.4)	32 (1.0)	89 (16.1)	30 (1.1)	216 (7.9)	98 (0.5)	14,851 (6.9)	11,791 (0.6)
Corpus uteri	806 (1.5)	13,394 (2.8)	813 (2.1)	14,910 (3.6)	1086 (2.4)	12,483 (3.3)	1227 (1.7)	23,921 (3.4)	6 (1.7)	117 (3.8)	6 (1.1)	17 (0.6)	66 (2.4)	523 (2.7)	4010 (1.9)	65,365 (3.3)
Ovary and tubes	1239 (2.3)	9241 (1.9)	1241 (3.2)	8684 (2.1)	1240 (2.8)	7260 (1.9)	1890 (2.6)	11,873 (1.7)	15 (4.2)	82 (2.6)	21 (3.8)	63 (2.3)	53 (1.9)	315 (1.6)	5699 (2.7)	37,518 (1.9)
Prostate	609 (1.2)	79,556 (16.7)	938 (2.4)	91,406 (22.3)	1003 (2.2)	86,215 (22.7)	1866 (2.5)	189,049 (26.8)	<5 (NA)	588 (18.9)	<5 (NA)	121 (4.3)	51 (1.9)	4121 (21.5)	4471 (2.1)	451,056 (22.6)
Kidney	1558 (3.0)	14,602 (3.1)	1293 (3.3)	15,186 (3.7)	1632 (3.7)	12,763 (3.4)	1929 (2.6)	19,413 (2.8)	16 (4.5)	140 (4.5)	16 (2.9)	102 (3.7)	101 (3.7)	950 (5.0)	6545 (3.1)	63,156 (3.2)
Melanoma of skin	11,959 (22.7)	28,881 (6.1)	5197 (13.2)	19,420 (4.7)	7284 (16.3)	25,770 (6.8)	13,276 (18.0)	47,103 (6.7)	67 (18.8)	87 (2.8)	15 (2.7)	26 (0.9)	355 (13.0)	587 (3.1)	38,153 (17.8)	121,874 (6.1)
Thyroid	2567 (4.9)	3591 (0.8)	3270 (8.3)	5494 (1.3)	2808 (6.3)	3845 (1.0)	4214 (5.7)	5659 (0.8)	16 (4.5)	18 (0.6)	14 (2.5)	17 (0.6)	228 (8.4)	284 (1.5)	13,117 (6.1)	18,908 (0.9)
Non Hodgkin lymphoma	2432 (4.6)	20,066 (4.2)	2266 (5.8)	18,970 (4.6)	1964 (4.4)	13,755 (3.6)	3183 (4.3)	26,263 (3.7)	16 (4.5)	131 (4.2)	28 (5.1)	83 (3.0)	126 (4.6)	755 (3.9)	10,015 (4.7)	80,023 (4.0)
Multiple myeloma	417 (0.8)	7827 (1.6)	302 (0.8)	6836 (1.7)	454 (1.0)	7227 (1.9)	623 (0.8)	12,310 (1.7)	<5 (NA)	54 (1.7)	<5 (NA)	35 (1.3)	31 (1.1)	387 (2.0)	1833 (0.9)	34,676 (1.7)
Leukaemia	1675 (3.2)	13,762 (2.9)	1287 (3.3)	9675 (2.4)	1530 (3.4)	9919 (2.6)	2552 (3.5)	18,691 (2.7)	14 (3.9)	84 (2.7)	11 (2.0)	21 (0.8)	82 (3.0)	516 (2.7)	7151 (3.3)	52,668 (2.6)
Total	52,799 (100)	476,988 (100)	39,264 (100)	409,426 (100)	44,581 (100)	379,007 (100)	73,671 (100)	705,298 (100)	356 (100)	3117 (100)	553 (100)	2787 (100)	2728 (100)	19,128 (100)	213,952 (100)	1,995,751 (100)

Data were suppressed to maintain anonymity if counts <5 (counts presented as <5 and rates as NA). All cells with <5 were in reality >0.

### Incidence trends comparing ages 20–49 to 50–84 years

**Fig. 1:**
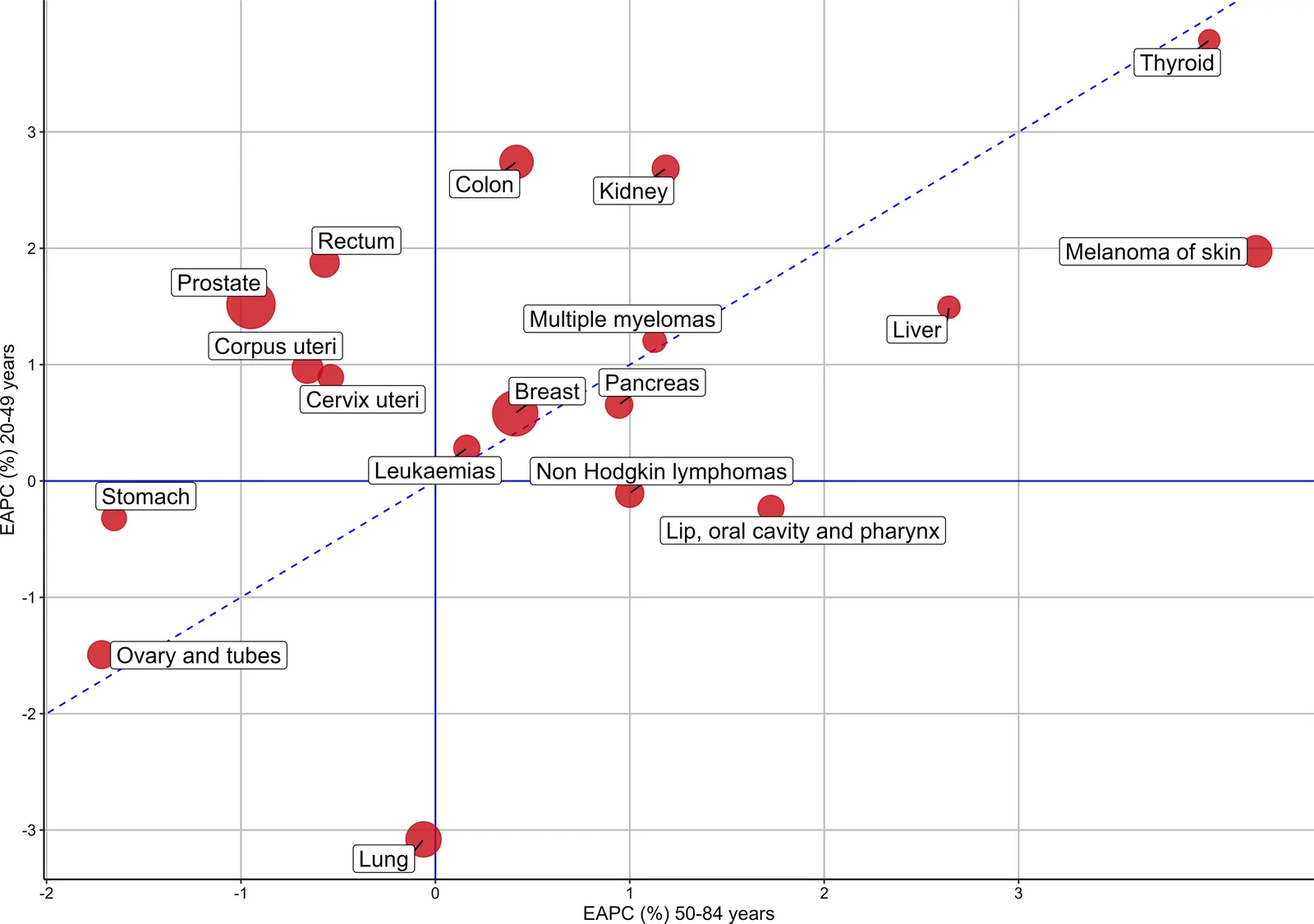
Estimated Annual Percent Change (EAPC) in age-standardised incidence rates per cancer site for the Nordic population combined between 2003 and 2022, for ages 20–49 and 50–84 years. Estimates are provided for men and women combined, except for cancers of the breast, ovary and tubes, cervix uteri, and corpus uteri (women-specific), and prostate (men-specific). The size of the bubbles is proportional to the crude incidence rate at ages 20–84 years during 2003–2022 for the given cancer site. The dashed line indicates where the EAPC at ages 20–49 and 50–84 years is equal. The plotted estimates, alongside 95% confidence intervals, are provided in Supplemental Table S3.

In pooled analyses across all Nordic countries and both sexes, significant increases in incidence rates with EAPCs ≥2.0% at younger ages (20–49 years) during 2003–2022 were observed for cancers of the thyroid, colon, kidney, and melanoma (Fig. 1, Supplemental Table S3). Early-onset incidence rates also increased for cancers of the rectum, prostate, liver, corpus uteri, as well as multiple myeloma (EAPC 1.0–1.9%), and for cancers of the cervix uteri, pancreas, and breast (EAPC 0.1–0.9%).

For most of these cancer sites, incidence rates also increased among individuals aged 50–84 years, but with lower EAPC compared with the younger age group. The exceptions were melanoma and liver cancer where the EAPCs were even higher at ages 50–84 years than at ages 20–49. Additionally, for cancers of the rectum, prostate, corpus uteri, and cervix uteri, incidence rates increased over time exclusively among the younger individuals, while simultaneously decreasing in the older age group.

Significantly decreasing early-onset incidence rates were also observed for lung cancer (EAPC −3.1%) and ovarian cancer (EAPC −1.5%). Non-significant declines were observed for non-Hodgkin lymphoma, stomach cancer, and cancers of the lip, oral cavity, and pharynx (EAPC ranging from −0.1 to −0.5%).

### Incidence trends by country

**Fig. 2:**
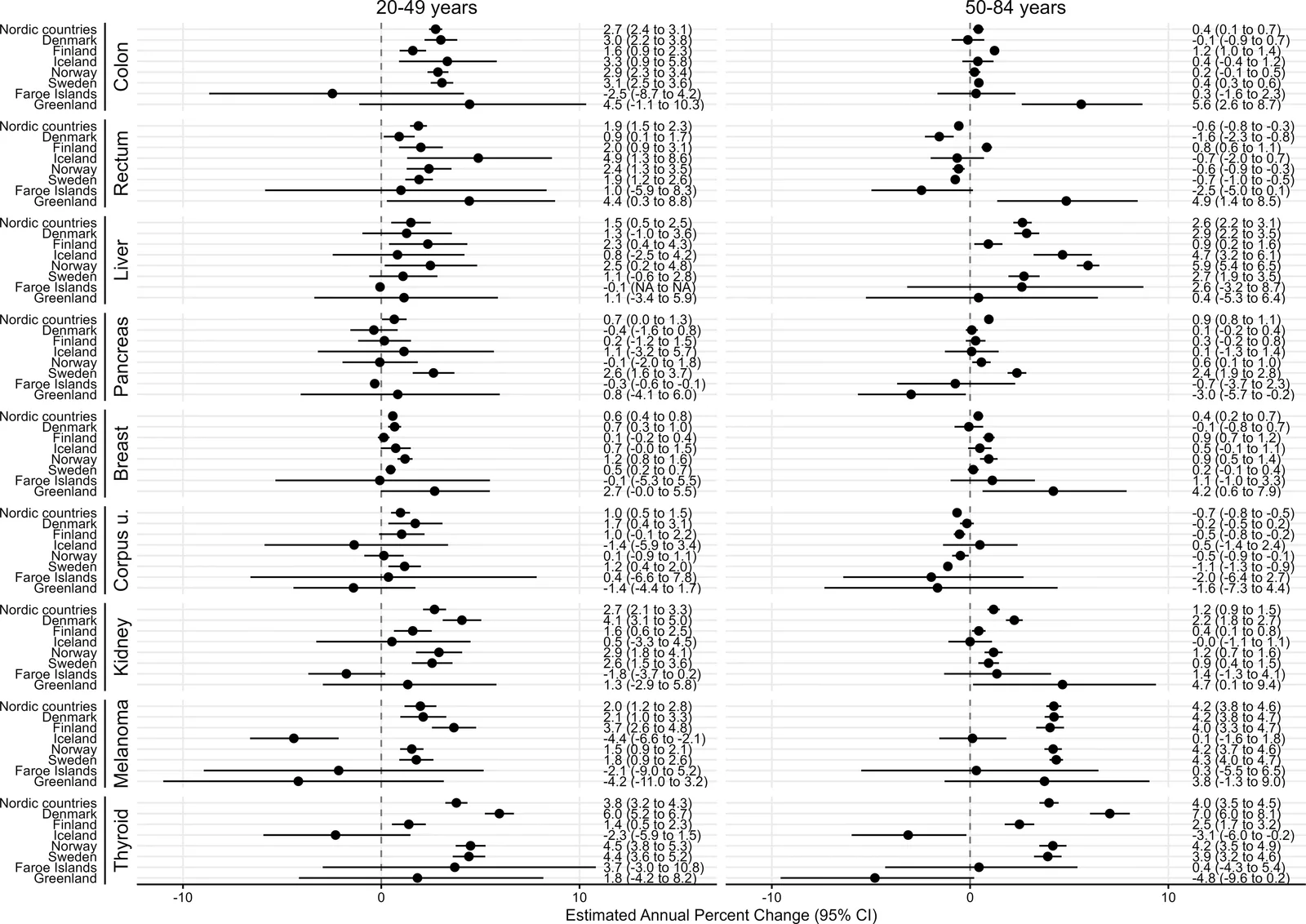
Estimated Annual Percent Change (EAPC) in age-standardised incidence rates for the nine selected cancer sites for which the EAPC at ages <50 years was significantly greater than zero in at least one 5-year age group. Results are shown by country for age ages 20–49 and 50–84 years. Estimates are provided for men and women combined, except for sex-specific cancers, breast, ovary and tubes, cervix uteri, corpus uteri and prostate.

For the nine cancer sites with significantly increasing incidence rates at ages 20–49 years in the pooled Nordic analysis, trends were similar in Denmark, Finland, Norway, and Sweden for melanoma, colon, rectal, female breast, kidney, and thyroid cancer (Fig. 2, left panel). In contrast, incidence patterns were less consistent in Iceland, Faroe Islands, and Greenland, particularly for melanoma. Sweden was the only country with an increasing incidence rate of early-onset pancreatic cancer.

For comparison, EAPCs per country and cancer sites are also presented for ages 50–84 years, revealing broadly similar incidence trends across countries, except for rectal, liver, pancreatic, and thyroid cancer (Fig. 2, right panel).

### Incidence trends by 5-year age groups

**Fig. 3:**
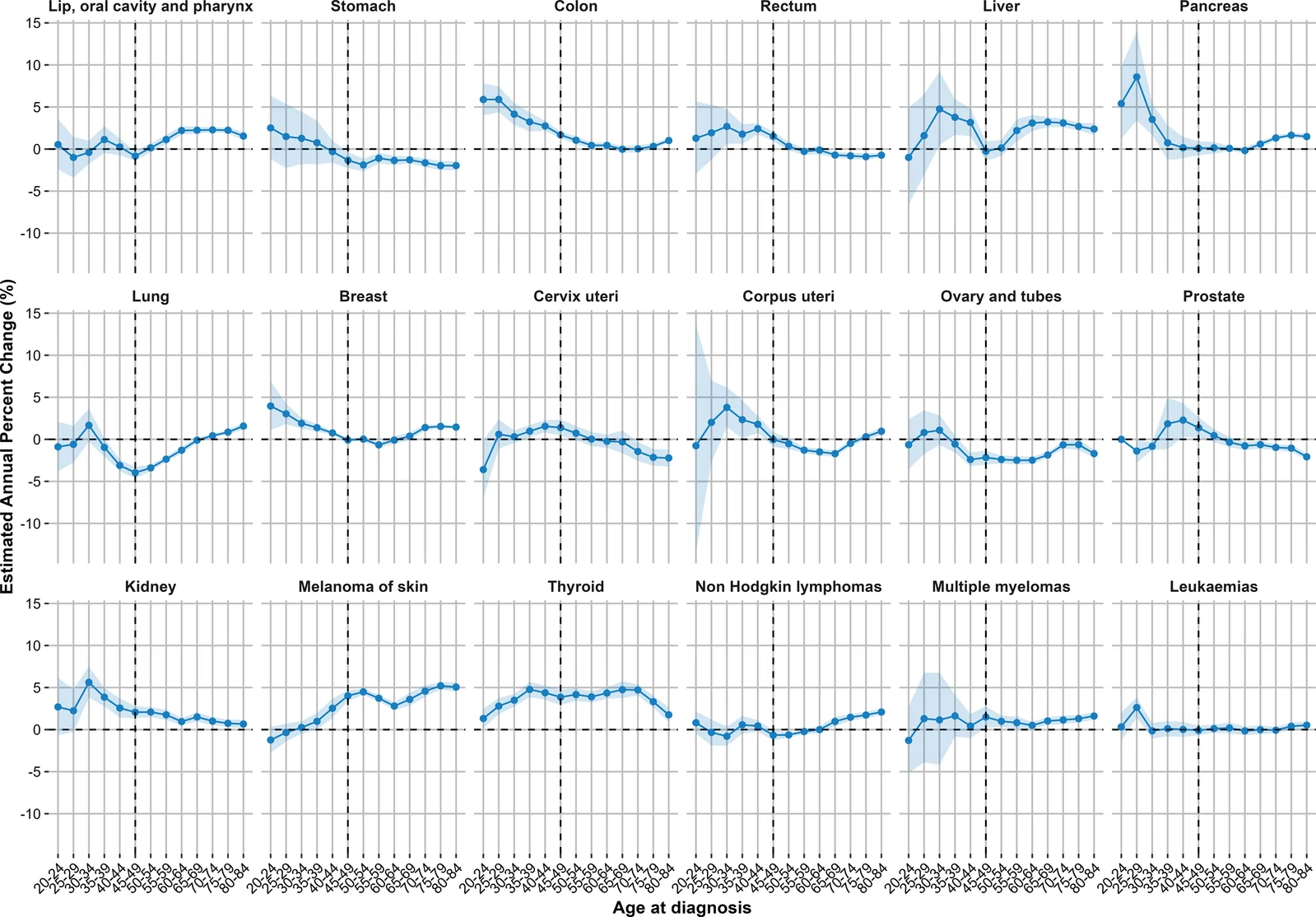
Age-specific Estimated Annual Percent Change (EAPC) in age-standardised incidence rates per cancer site for Nordic population combined between 2003 and 2022. Estimates are provided for men and women combined, except for sex-specific cancers: breast, ovary and tubes, cervix uteri, corpus uteri and prostate.

Analyses stratified by 5-year age groups showed strong age gradients in EAPCs. The largest increases were observed at the youngest ages (<35 years) for cancers of the colon, pancreas, breast, and kidney, while increases were largest at ages 35–49 years for cancers of the rectum, liver, corpus uteri, thyroid, and melanoma (Fig. 3). For melanoma, the increasing early-onset trend was limited to 2003–2012, followed by a decreasing trend during 2013–2022 (Supplemental Figure S1). Cancer sites with no significant increases in trends within any 5-year age group between 20 and 49 years included lip, oral cavity and pharynx, multiple myelomas, non-Hodgkin lymphoma, prostate, and stomach, while the rates declined for lung and ovarian cancer at ages 35–49 years (Fig. 3). Minor increases were observed for cervical cancer and leukaemia. For prostate cancer, a decline in incidence rates was observed during 2013–2022 (Supplemental Figure S1).

The incidence trends were similar in men and women, with the exceptions of liver and pancreatic cancer for which significant increases in early-onset trends were confined to men only and women only, respectively (Supplemental Figures S2 and S3).

### Age-specific incidence trends by calendar period (since 1978) and birth cohort

**Fig. 4:**
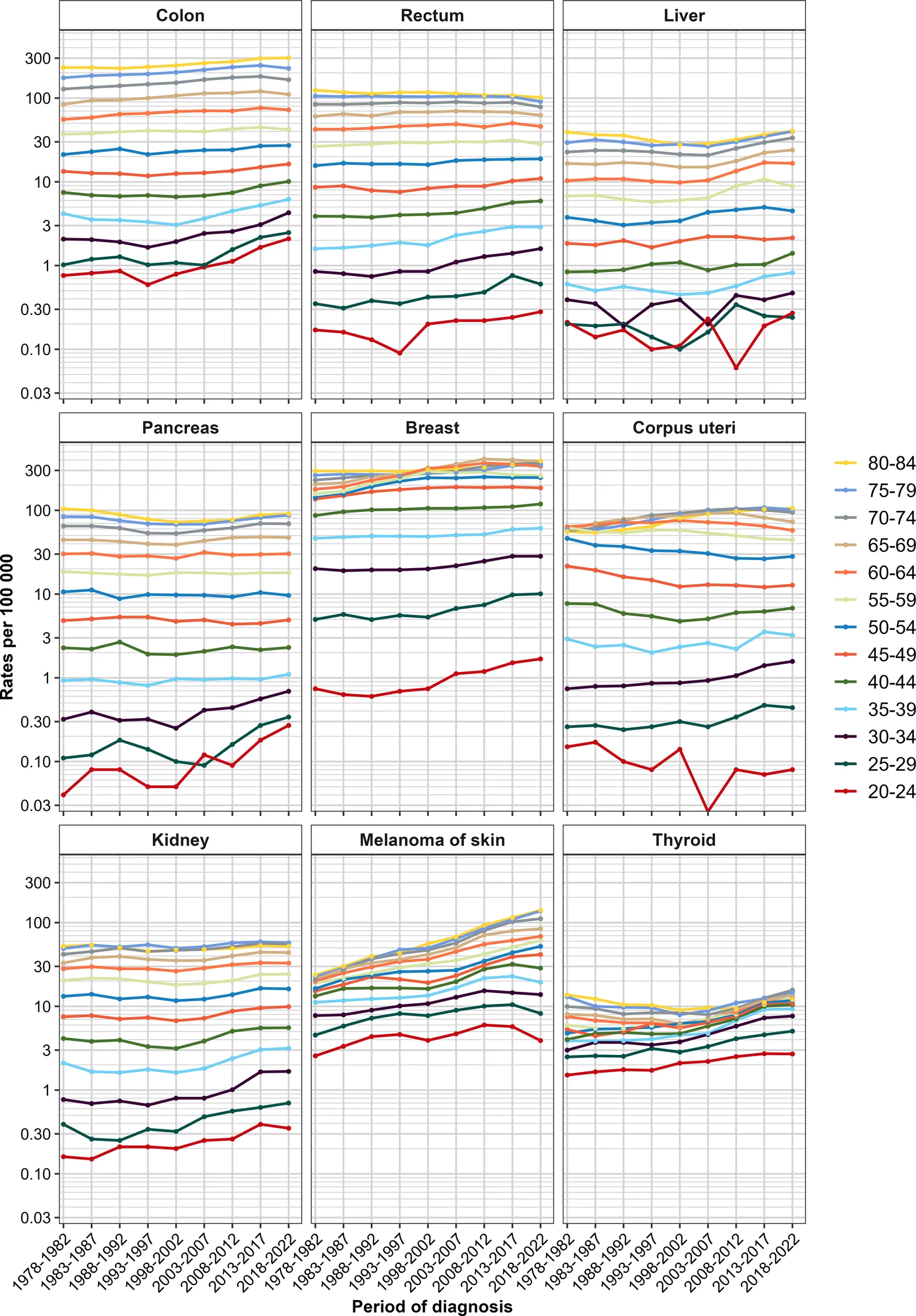
Age-specific incidence trends across period of diagnosis (1978–2022), by cancer site, for the Nordic population combined, for the nine selected cancer sites for which the EAPC at ages <50 years was significantly greater than zero in at least one 5-year age group.

Focussing on the nine cancer sites with the largest recent increases in early-onset incidence, we compared incidence trends by age across calendar period from 1978 onwards (Fig. 4). For colon cancer, the incidence rates increased among individuals <40 years since the 1990s, whereas the incidence in older age groups decreased in the most recent decade. For rectal cancer, the early-onset incidence rates also increased, although to a lesser extent than for colon cancer, whereas liver cancer incidence rates appeared to have increased across most age groups in recent years.

Pancreatic cancer exhibited larger incidence increases in individuals <35 years. Breast cancer incidence rates increased steadily in women aged <50 years from the early 1990s onwards. For corpus uteri cancer, increasing incidence was observed in women aged 25–44 years in recent years. Kidney cancer incidence rates increased in individuals <50 years in the 2000s, followed by a plateau in 2013–2022. Melanoma incidence increased across all age groups from the early 1980s, then plateaued in the beginning of the most recent decade (2013–2022), and subsequently declined in individuals younger than 45 years. Thyroid cancer incidence rates increased since the early 2000s across all age groups.

**Fig. 5:**
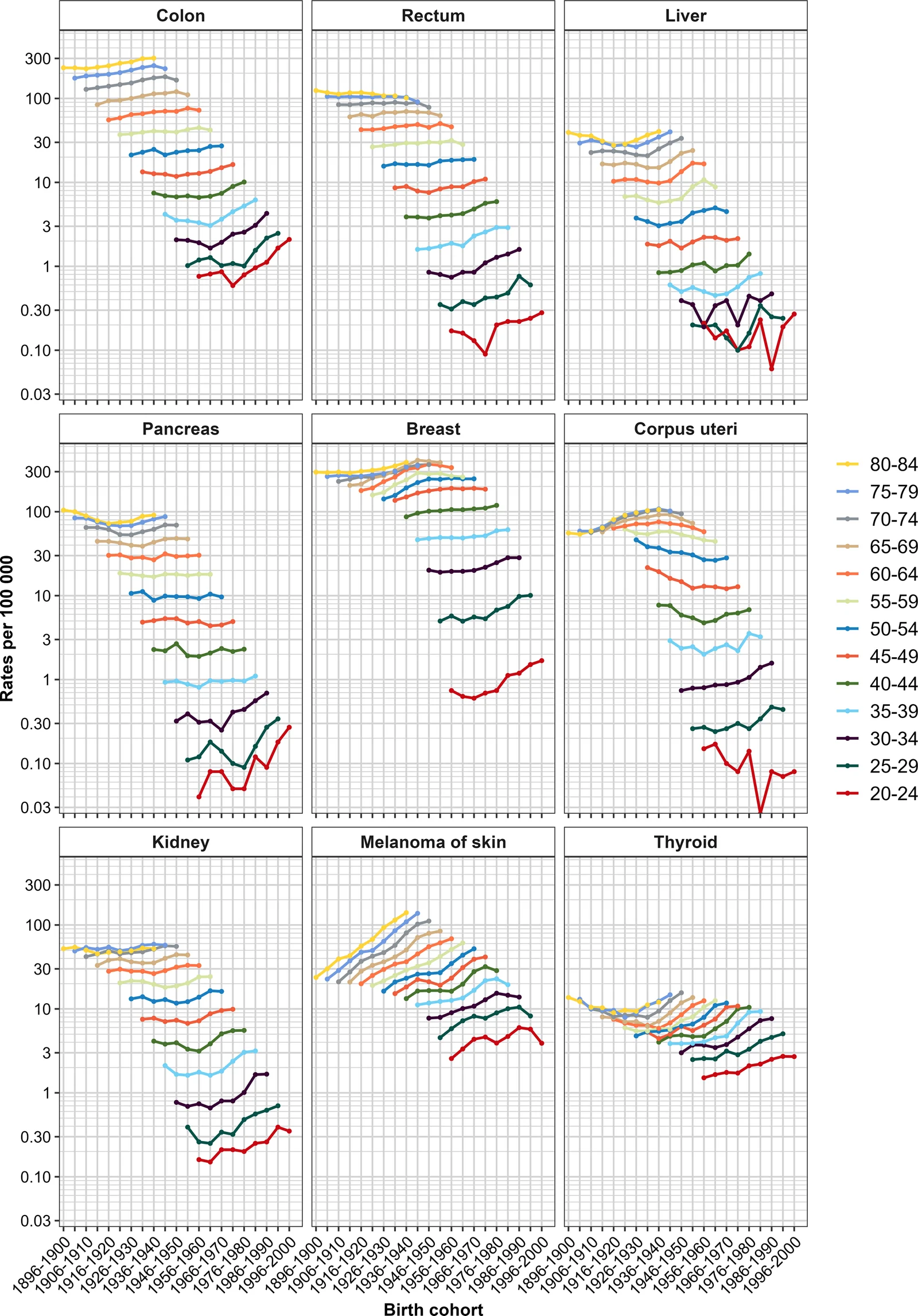
Age-specific incidence trends across birth cohort (1896–2000), by cancer site, for the Nordic population combined, for the nine selected cancer sites for which the EAPC at ages <50 years was significantly greater than zero in at least one 5-year age group.

Fig. 5 shows the age-specific incidence rates by birth cohort. For several cancer sites, the increase in early-onset incidence rates were confined to the youngest birth cohorts. Successive cohorts born from approximately 1960 onward had higher rates of colon cancer compared to those born earlier, suggesting that younger generations are at increasingly higher risk of being diagnosed with the disease relative to their predecessors. Similarly, increased incidence rates in more recent birth cohorts were found for early-onset rectal, pancreatic, breast, and kidney cancer from 1970s onwards.

For completeness, the age-specific incidence rates by period and birth cohort are presented for the remaining nine cancer sites (lip, oral cavity and pharynx; stomach; lung; cervix uteri; ovary and tubes; prostate; non-Hodgkin lymphoma; multiple myeloma; and leukaemias) in Supplemental Figures S4 and S5.

### Changes in absolute burden of early-onset cancer

Comparing the first (2003–2007) and last (2018–2022) time periods under study, the total number of early-onset cases across the 18 selected cancer sites increased by 9174 (from 47,491 to 56,665) compared to 145,125 (from 423,087 to 568,212) at ages 50–84 years (Supplemental Table S4). At ages 20–49 years, the largest absolute increase was observed for melanoma, with 2918 additional cases (7306–10,224). The corresponding increases were 1800 cases for thyroid cancer (2279–4079), 1641 for breast cancer (15,891–17,532), 1298 for colon cancer (2360–3658), and 650 for kidney cancer (1232–1882).

## Discussion

This is the first study to comprehensively characterise the increasing, yet heterogenous, burden of early-onset cancer across the Nordic countries. We observed increasing early-onset incidence rates for 12 of 18 major sites, which may reflect both shifts in the exposure to early-life risk factors and changes in diagnostic measures and intensity over time. The most pronounced increases occurred for thyroid, colon, and kidney cancer, as well as melanoma. For colon and kidney cancers, the increases were markedly higher among younger than older individuals, while for thyroid cancer equally large increases were observed across age groups. In contrast, early-onset melanoma incidence decreased during the most recent decade under study. For rectal, prostate, and corpus uteri cancer, the increasing trends in the younger ages diverged from decreasing rates in the older age group. Among the six sites with non-increasing early-onset incidence rates, the largest decreases occurred for lung and ovarian cancer. In comparison with historical trends, the increasing early-onset cancer rates were primarily concentrated to the two most recent decades (2003–2022) and in birth cohorts from 1980s onwards. For colon cancer, the early-onset rates increased in successive birth cohorts from the 1960s, and for pancreas, thyroid, and breast, as well as melanoma, since the late 1970s. During the most recent decade (2013–2022), the increases were particularly steep for colon and pancreatic cancer. The rising incidence rates of several early-onset cancers highlights the need for an increased awareness among clinicians and the public, and underscores the importance of further research to improve the understanding of the underlying aetiologic factors and biological mechanisms.

Our results are broadly consistent with results from other settings, yet we found notable site-specific differences in both the direction and the magnitude of early-onset incidence trends in the Nordic countries. An international comparison including 41 countries reported increases in early-onset colon cancer in several countries, especially in the U.S., Canada, Japan, and Korea, but not in all European countries.^3^ The same study reported increases in early-onset kidney cancer in some countries, and more often in men, while increases in early-onset breast cancer were found in several European and Asian countries, but not in the U.S. Another international comparative analysis, including five Nordic countries, found overall similar trends to those reported here; however, it was based on data from 2003 to 2017 only.^6^ Studies from the U.S. have shown substantial increases in early-onset cancers of the colon, rectum, corpus uteri, kidney, pancreas, and thyroid, as well as myeloma, in line with our findings.^2^^,^^5^ In a recent study, increasing incidence rates of early-onset breast and liver cancer were also observed in the U.S. between 2010 and 2019.^4^ In nearly all countries, including the Nordic countries, marked increases in thyroid cancer incidence rates have been observed in both younger and older individuals.^3^

These findings are likely to reflect shifts in risk factor exposure in successive generations (e.g., obesity, diet, physical activity, smoking, alcohol consumption, UV radiation, and reproductive factors).^1^^,^^7^, ^8^, ^9^ In line with our findings, several studies have reported increasing incidence of early-onset obesity-related cancers, particularly in Western countries.^2^, ^3^, ^4^, ^5^^,^^20^^,^^21^ In our study, we found increasing incidence for seven of the nine obesity-related sites defined by the WHO,^22^ including colon, rectum, liver, pancreas (mainly driven by an increase in Sweden), corpus uteri, kidney, and thyroid cancer, but not for stomach and ovarian cancer. However, we found large differences across obesity-associated cancers, which were to some extent inconsistent with previously reported strengths of associations between obesity and cancer. This suggests that the increasing incidences cannot be attributed to obesity alone. Furthermore, obesity is related to dietary habits (e.g., high intake of fat, sugar, red meat, and low food fibre intake) and sedentary lifestyle (low physical activity), which represent independent risk factors for several cancers.^7^ It is known that dietary habits have changed across generations, and the prevalences of obesity and sedentary lifestyle are increasing in younger generations and will likely influence future disease development, including cancer, at older ages.^14^^,^^23^^,^^24^ Smoking and alcohol consumption are established risk factors for colorectal and pancreatic cancer; however, the prevalence of these exposures has decreased among the younger generations in the Nordic countries, potentially mitigating the impact of changes in the exposure to other risk factors, including obesity and dietary habits.^7^^,^^14^

The increases in early-onset cancer in successive generations since 1980s onwards suggest that these generations may have been exposed to higher levels of carcinogenic factors during early life.^2^^,^^5^^,^^25^ It has also been suggested that accelerated ageing, assessed by the difference between biological and chronological age, may be more prevalent in recent generations^26^ and contributing to an increase of early-onset cancer. The hypothesis is of interest since biological age is likely to capture the cumulative impact of lifestyle and early life exposures, is potentially modifiable and may open up venues for primary prevention. Advanced biological age has been associated with increased risk of overall cancer, as well as lung, and colorectal cancer.^27^ However, given the ecological and descriptive nature of this study, it remains unknown to what extent exposure to the abovementioned risk factors in early life and adulthood contribute to the observed increase in early-onset cancer incidence in the Nordic countries. This highlights the need for studies with individual-level data on risk factors, including obesity and other lifestyle factors.

Compared to other geographic regions, melanoma incidence is high in the Nordic countries.^28^ The increasing incidence trends among young and old individuals may reflect both changes in UV exposure and increased diagnostic scrutiny, including lower thresholds for biopsying pigmented lesions.^29^ Although we found an overall increase in early-onset melanoma (2003–2022), the decline among the younger birth cohorts in the most recent decade may reflect an impact of improved sun-protective behaviours (e.g., avoidance of direct sunlight, increased use of sunscreen products, and restricted access to tanning salons) in the Nordic countries, in particular during childhood and adolescence.^30^ Sun protection awareness campaigns have been undertaken in the Nordic countries in the 2000s.^31^

In line with international trends, reproductive patterns in the Nordic countries have shifted substantially, with fewer childbirths per woman and later age at first birth.^32^ While high parity confers protection against postmenopausal breast cancer, the role of childbearing in premenopausal women is more complex. Shifts in reproductive patterns are therefore unlikely to explain the increase in early onset breast cancer in women <35 years.^33^ All Nordic countries, except Greenland, have implemented organised screening programmes for breast cancer, albeit with different starting times and age-groups included. Sweden and Iceland offer breast cancer screening for ages 40–74 years, which may have contributed to the observed trends in both early- and late-onset incidence, while in Denmark, Norway, Finland, and the Faroe Islands, the target age range is 50–69 years only. Also, we cannot rule out use of opportunistic screening outside of the screening ages and its potential contribution to increasing incidence rates of breast cancer in younger and older women.^34^ Organised cervical cancer screening is available from age 23 or 25 (age 30 in Finland) years in all countries including Greenland. The reasons for the decreasing incidence rates of early-onset ovarian cancer remain largely unknown, yet may reflect changes in use of hormonal contraceptives.^35^, ^36^, ^37^, ^38^

Incidence trends are also likely to have been affected by increased awareness combined with more frequent use of sensitive diagnostic tools such as ultrasound, computed tomography, and magnetic resonance imaging.^9^ The changes in diagnostic procedures over time, with increasingly sensitive diagnostic methods are likely to have contributed primarily to earlier detection of thyroid, kidney, liver, pancreatic, and prostate cancer across most age groups, although potentially to a lesser extent among the oldest individuals. The substantial global increase in thyroid cancer has, in particular, been attributed to increases in diagnostic intensity.^3^^,^^8^^,^^39^^,^^40^ A likely explanation for the increase in early-onset prostate cancer is opportunistic prostate-specific antigen testing in men under age 50 years in both the U.S. and the Nordic countries, despite the absence of formal recommendations from national health authorities.^41^^,^^42^

The increasing incidence rates of early-onset cancer calls for strengthened preventive strategies.^1^ This is especially important given that younger generations experiencing elevated cancer risk are likely to carry this excess risk into older ages when cancer becomes more common.^5^ Although the absolute increase in early-onset cancer in the Nordic countries has been relatively modest (an estimated 9174 additional cases between 2003–2007 and 2018–2022 for the 18 selected cancer sites), the observed upward trend underscores the importance of focused primary cancer prevention, starting at an early age, and increased awareness. Furthermore, as younger patients are likely to experience prolonged survivorship and cancer-related morbidity over the life course, more healthcare resources tailored to younger cancer patients will be needed in future.

A major strength of the present study was the use of the NORDCAN database comprising population-based, individual-level cancer registry data from seven Nordic countries with comparable tax-funded healthcare systems and a combined population of nearly 28 million in 2022. Although the Nordic cancer registries are generally characterised by high completeness and validity, there are some differences in registration routines and inclusion criteria for certain tumour entities, and we therefore excluded brain, bladder, and non-melanoma skin cancer.^17^ Because death certificate-initiated cancers are not included by the Swedish Cancer Registry, inter-country comparisons of absolute incidence rates may have been affected for Sweden, particularly in the oldest age groups where routine reporting of cancers with a poor prognosis is less complete.^43^ Furthermore, similar to other registry-based studies exploring incidence trends, no information was available on individual-level history of risk factor exposures, as well as race or ethnicity. As the analysis included a large number of comparisons, false positive findings cannot be excluded. Finally, during the COVID-19 pandemic fewer cancers were diagnosed, particularly in Q2 2020 and, to a lesser extent, in Q1 2021.^44^ While there was some temporary delay in cancer diagnosis, it is unlikely to have considerably affected the overall long-term incidence trends presented here.

### Conclusion

Consistent with findings from other geographical regions, we found increasing incidence rates of early-onset cancer for several major sites across the Nordic countries. The most pronounced increases were observed for obesity-related malignancies, particularly thyroid, colon, and kidney cancers. In contrast, we observed decreases in early-onset incidence rates of lung and ovarian cancer.

Suggested explanations for these trends include changes in exposure to risk factors during early life and young adulthood. New diagnostic methods and increased diagnostic intensity are also likely to have contributed to the increase of early-onset cancer. Although the underlying mechanisms require further investigation, our findings underscore the importance of primary prevention strategies aimed at reducing exposure to established and suspected risk factors, along with strengthened research into identifying modifiable risk factors of early-onset cancer and increased awareness of malignant disease in young individuals not traditionally perceived as being at risk of developing cancer.

## Contributors

ALVJ, ML, SMK, and SF conceptualised the study. ALVJ and SMK contributed to the methodology. SMK carried out the analyses. ALVJ and ML drafted the manuscript. HM, GU, AS, ÅP, HB, LK, TT, EDDO, FB, RC, SMK, SF, and ML critically revised and edited the manuscript. ALVJ and SMK had full access to the data, verified the data, and take responsibility for the integrity and accuracy of the analyses. All authors had full access to all the findings reported in the manuscript and accept responsibility to submit the paper for publication.

## Data sharing statement

The data used for this study can be found at https://nordcan.iarc.fr/en.

## Declaration of interests

RC was employed as a Senior Editor at The Lancet Regional Health—Europe from 1st April 2024 to 30th September 2025. RC was not involved in any stage of the peer review process for this Article. ML owns stocks in Astra Zeneca and Pfizer Inc. The other authors declare no conflict of interest. Where authors are identified as personnel of the International Agency for Research on Cancer/WHO, the authors alone are responsible for the views expressed in this article and they do not necessarily represent the decisions, policy, or views of the International Agency for Research on Cancer/WHO.
