# Supplementary material for: Trends in early-onset cancer incidence rates in the Nordic countries (2003–2022): a population-based study
Source: Lancet Reg Health Eur. 2026 Sep 12;70:101858. doi: 10.1016/j.lanepe.2026.101858 (PMC13587733; doi:10.1016/j.lanepe.2026.101858)

SUPPLEMENTAL MATERIAL

Supplemental Table S1. The Nordic population (NORDCAN) in year 2000 used for calculation of age-standardised rates (ASR), the number of persons and proportion per 5-year age group and 100 000 inhabitants.

Age (years)	Nordic ASR (N)	Proportion
0-4	5900	5.9%
5-9	6600	6.6%
10-14	6200	6.2%
15-19	5800	5.8%
20-24	6100	6.1%
25-29	6800	6.8%
30-34	7300	7.3%
35-39	7300	7.3%
40-44	7000	7.0%
45-49	6900	6.9%
50-54	7400	7.4%
55-59	6100	6.1%
60-64	4800	4.8%
65-69	4100	4.1%
70-74	3900	3.9%
75-79	3500	3.5%
80-84	2400	2.4%
85+	1900	1.9%
Total	100000	100.0%

In NORDCAN online, additional age standards for World, European 2013 and European 1976 are also available (https://nordcan.iarc.fr/en/additional-information?tab=2).

Supplemental Table S2. Number of cancer cases by country across 18 cancer sites for men and women separately at ages 20-49 and 50-84 years in 2003-2022.

Cancer sites	Denmark		Finland		Norway		Sweden		Faroe Islands		Greenland		Iceland		Nordic countries	
	20-49	50-84	20-49	50-84	20-49	50-84	20-49	50-84	20-49	50-84	20-49	50-84	20-49	50-84	20-49	50-84
	N (%)	N (%)	N (%)	N (%)	N (%)	N (%)	N (%)	N (%)	N (%)	N (%)	N (%)	N (%)	N (%)	N (%)	N (%)	N (%)
**Men**																
Lip, oral cavity and pharynx	1135 (7.8)	11131 (4.5)	763 (6.9)	6458 (2.9)	646 (5.2)	5986 (2.8)	1157 (5.9)	11064 (2.8)	8 (9.2)	60 (3.6)	27 (17.8)	150 (10.2)	57 (8.3)	256 (2.6)	3793 (6.5)	35105 (3.2)
Stomach	481 (3.3)	7120 (2.9)	422 (3.8)	6472 (2.9)	321 (2.6)	4874 (2.3)	564 (2.9)	9063 (2.3)	5 (5.7)	52 (3.1)	18 (11.8)	105 (7.1)	20 (2.9)	261 (2.6)	1831 (3.1)	27947 (2.6)
Colon	1230 (8.4)	25956 (10.5)	1076 (9.8)	16039 (7.3)	1388 (11.1)	21656 (10.3)	2003 (10.1)	34711 (8.8)	6 (6.9)	204 (12.3)	14 (9.2)	210 (14.3)	80 (11.6)	1025 (10.2)	5797 (9.9)	99801 (9.2)
Rectum	859 (5.9)	16119 (6.5)	610 (5.5)	11771 (5.3)	929 (7.4)	12965 (6.1)	1257 (6.4)	21103 (5.4)	<5 (NA)	122 (7.3)	18 (11.8)	135 (9.2)	42 (6.1)	390 (3.9)	3718 (6.3)	62605 (5.8)
Liver	210 (1.4)	5163 (2.1)	186 (1.7)	5797 (2.6)	214 (1.7)	2652 (1.3)	418 (2.1)	7954 (2.0)	<5 (NA)	27 (1.6)	5 (3.3)	39 (2.7)	10 (1.5)	233 (2.3)	1044 (1.8)	21865 (2.0)
Pancreas	424 (2.9)	9035 (3.7)	381 (3.5)	9994 (4.5)	318 (2.5)	6561 (3.1)	489 (2.5)	11333 (2.9)	<5 (NA)	89 (5.4)	11 (7.2)	71 (4.8)	20 (2.9)	340 (3.4)	1646 (2.8)	37423 (3.4)
Lung	1146 (7.8)	42629 (17.3)	671 (6.1)	31219 (14.2)	781 (6.3)	28574 (13.5)	877 (4.4)	35831 (9.1)	<5 (NA)	218 (13.1)	24 (15.8)	472 (32.1)	54 (7.9)	1462 (14.6)	3556 (6.0)	140405 (12.9)
Prostate	609 (4.2)	79556 (32.2)	938 (8.5)	91406 (41.5)	1003 (8.0)	86215 (40.8)	1866 (9.5)	189049 (48.0)	<5 (NA)	588 (35.4)	<5 (NA)	121 (8.2)	51 (7.4)	4121 (41.1)	4471 (7.6)	451056 (41.6)
Kidney	1090 (7.4)	9756 (4.0)	863 (7.8)	9045 (4.1)	1101 (8.8)	8636 (4.1)	1302 (6.6)	12384 (3.1)	10 (11.5)	88 (5.3)	6 (3.9)	69 (4.7)	70 (10.2)	594 (5.9)	4442 (7.6)	40572 (3.7)
Melanoma of skin	4160 (28.4)	15051 (6.1)	2117 (19.2)	10979 (5.0)	2804 (22.5)	13923 (6.6)	5149 (26.1)	25914 (6.6)	14 (16.1)	42 (2.5)	<5 (NA)	16 (1.1)	111 (16.2)	290 (2.9)	14360 (24.4)	66215 (6.1)
Thyroid	629 (4.3)	1202 (0.5)	699 (6.3)	1668 (0.8)	651 (5.2)	1301 (0.6)	901 (4.6)	1845 (0.5)	6 (6.9)	6 (0.4)	<5 (NA)	<5 (NA)	42 (6.1)	103 (1.0)	2930 (5.0)	6128 (0.6)
Non Hodgkin lymphoma	1465 (10.0)	11323 (4.6)	1365 (12.4)	10175 (4.6)	1162 (9.3)	7745 (3.7)	1879 (9.5)	15008 (3.8)	12 (13.8)	76 (4.6)	16 (10.5)	47 (3.2)	71 (10.3)	414 (4.1)	5970 (10.2)	44788 (4.1)
Multiple myeloma	224 (1.5)	4489 (1.8)	178 (1.6)	3597 (1.6)	277 (2.2)	4159 (2.0)	375 (1.9)	7129 (1.8)	<5 (NA)	31 (1.9)	<5 (NA)	18 (1.2)	12 (1.7)	217 (2.2)	1068 (1.8)	19640 (1.8)
Leukaemia	970 (6.6)	8306 (3.4)	741 (6.7)	5714 (2.6)	890 (7.1)	5991 (2.8)	1503 (7.6)	11287 (2.9)	11 (12.6)	58 (3.5)	5 (3.3)	13 (0.9)	47 (6.8)	331 (3.3)	4167 (7.1)	31700 (2.9)
Total	14632 (100)	246836 (100)	11010 (100)	220334 (100)	12485 (100)	211238 (100)	19740 (100)	393675 (100)	87 (100)	1661 (100)	152 (100)	1469 (100)	687 (100)	10037 (100)	58793 (100)	1085250 (100)
**Women**																
Lip, oral cavity and pharynx	596 (1.6)	4767 (2.1)	496 (1.8)	3667 (1.9)	443 (1.4)	2985 (1.8)	845 (1.6)	6461 (2.1)	5 (1.9)	20 (1.4)	19 (4.7)	60 (4.6)	15 (0.7)	144 (1.6)	2419 (1.6)	18104 (2.0)
Stomach	328 (0.9)	3313 (1.4)	370 (1.3)	4215 (2.2)	290 (0.9)	2761 (1.6)	507 (0.9)	5268 (1.7)	<5 (NA)	28 (1.9)	10 (2.5)	44 (3.3)	20 (1.0)	171 (1.9)	1528 (1.0)	15800 (1.7)

Colon	1312 (3.4)	24592 (10.7)	1148 (4.1)	15140 (8.0)	1510 (4.7)	21975 (13.1)	2000 (3.7)	33650 (10.8)	16 (5.9)	176 (12.1)	16 (4.0)	210 (15.9)	87 (4.3)	861 (9.5)	6089 (3.9)	96604 (10.6)
Rectum	703 (1.8)	9617 (4.2)	585 (2.1)	7525 (4.0)	823 (2.6)	8414 (5.0)	1010 (1.9)	13338 (4.3)	15 (5.6)	106 (7.3)	17 (4.2)	82 (6.2)	46 (2.3)	278 (3.1)	3199 (2.1)	39360 (4.3)
Liver	142 (0.4)	2010 (0.9)	110 (0.4)	2637 (1.4)	115 (0.4)	1339 (0.8)	217 (0.4)	3797 (1.2)	<5 (NA)	21 (1.4)	<5 (NA)	18 (1.4)	11 (0.5)	83 (0.9)	598 (0.4)	9905 (1.1)
Pancreas	345 (0.9)	8071 (3.5)	317 (1.1)	9460 (5.0)	302 (0.9)	5784 (3.4)	457 (0.8)	10940 (3.5)	<5 (NA)	59 (4.1)	10 (2.5)	72 (5.5)	12 (0.6)	311 (3.4)	1445 (0.9)	34697 (3.8)
Lung	1452 (3.8)	40931 (17.8)	491 (1.7)	15735 (8.3)	892 (2.8)	23823 (14.2)	1186 (2.2)	36909 (11.8)	5 (1.9)	171 (11.7)	20 (5.0)	374 (28.4)	63 (3.1)	1640 (18.0)	4109 (2.6)	119583 (13.1)
Breast	15267 (40.0)	72471 (31.5)	13371 (47.3)	71232 (37.7)	12894 (40.2)	46802 (27.9)	24696 (45.8)	105209 (33.8)	86 (32.0)	431 (29.6)	139 (34.7)	230 (17.5)	882 (43.2)	3137 (34.5)	67335 (43.4)	299512 (32.9)
Cervix uteri	3907 (10.2)	3143 (1.4)	1660 (5.9)	1484 (0.8)	3714 (11.6)	2619 (1.6)	5228 (9.7)	4385 (1.4)	37 (13.8)	32 (2.2)	89 (22.2)	30 (2.3)	216 (10.6)	98 (1.1)	14851 (9.6)	11791 (1.3)
Corpus uteri	806 (2.1)	13394 (5.8)	813 (2.9)	14910 (7.9)	1086 (3.4)	12483 (7.4)	1227 (2.3)	23921 (7.7)	6 (2.2)	117 (8.0)	6 (1.5)	17 (1.3)	66 (3.2)	523 (5.8)	4010 (2.6)	65365 (7.2)
Ovary and tubes	1239 (3.2)	9241 (4.0)	1241 (4.4)	8684 (4.6)	1240 (3.9)	7260 (4.3)	1890 (3.5)	11873 (3.8)	15 (5.6)	82 (5.6)	21 (5.2)	63 (4.8)	53 (2.6)	315 (3.5)	5699 (3.7)	37518 (4.1)
Kidney	468 (1.2)	4846 (2.1)	430 (1.5)	6141 (3.2)	531 (1.7)	4127 (2.5)	627 (1.2)	7029 (2.3)	6 (2.2)	52 (3.6)	10 (2.5)	33 (2.5)	31 (1.5)	356 (3.9)	2103 (1.4)	22584 (2.5)
Melanoma of skin	7799 (20.4)	13830 (6.0)	3080 (10.9)	8441 (4.5)	4480 (14.0)	11847 (7.1)	8127 (15.1)	21189 (6.8)	53 (19.7)	45 (3.1)	10 (2.5)	10 (0.8)	244 (12.0)	297 (3.3)	23793 (15.3)	55659 (6.1)
Thyroid	1938 (5.1)	2389 (1.0)	2571 (9.1)	3826 (2.0)	2157 (6.7)	2544 (1.5)	3313 (6.1)	3814 (1.2)	10 (3.7)	12 (0.8)	12 (3.0)	14 (1.1)	186 (9.1)	181 (2.0)	10187 (6.6)	12780 (1.4)
Non Hodgkin lymphoma	967 (2.5)	8743 (3.8)	901 (3.2)	8795 (4.7)	802 (2.5)	6010 (3.6)	1304 (2.4)	11255 (3.6)	<5 (NA)	55 (3.8)	12 (3.0)	36 (2.7)	55 (2.7)	341 (3.8)	4045 (2.6)	35235 (3.9)
Multiple myeloma	193 (0.5)	3338 (1.5)	124 (0.4)	3239 (1.7)	177 (0.6)	3068 (1.8)	248 (0.5)	5181 (1.7)	<5 (NA)	23 (1.6)	<5 (NA)	17 (1.3)	19 (0.9)	170 (1.9)	765 (0.5)	15036 (1.7)
Leukaemia	705 (1.8)	5456 (2.4)	546 (1.9)	3961 (2.1)	640 (2.0)	3928 (2.3)	1049 (1.9)	7404 (2.4)	<5 (NA)	26 (1.8)	6 (1.5)	8 (0.6)	35 (1.7)	185 (2.0)	2984 (1.9)	20968 (2.3)
Total	38167 (100)	230152 (100)	28254 (100)	189092 (100)	32096 (100)	167769 (100)	53931 (100)	311623 (100)	269 (100)	1456 (100)	401 (100)	1318 (100)	2041 (100)	9091 (100)	155159 (100)	910501 (100)

Data were suppressed to maintain anonymity if counts <5 (counts presented as <5 and rates as NA). All cells with <5 were in reality >0, except for one in Greenland which was 0.

Supplemental Table S3. Estimated Annual Percent Change (EAPC) for ages 20-49 and 50-84 years per cancer sites, Nordic combined population 2003-2022, men and women combined (except for sex-specific cancers, breast, ovary and tubes, cervix uteri, corpus uteri and prostate). These estimates are plotted in Figure 1.

**Cancer sites**	**20-49**	**50-84**
	**EAPC % (95% CI)**	**EAPC % (95% CI)**
Lip, oral cavity and pharynx	-0.2 (-0.6, 0.1)	1.7 (1.5, 2.0)
Stomach	-0.3 (-1.1, 0.5)	-1.7 (-2, -1.3)
Colon	2.7 (2.4, 3.1)	0.4 (0.1, 0.7)
Rectum	1.9 (1.5, 2.3)	-0.6 (-0.8, -0.3)
Liver	1.5 (0.5, 2.5)	2.6 (2.2, 3.1)
Pancreas	0.7 (0.0, 1.3)	0.9 (0.8, 1.1)
Lung	-3.1 (-3.6, -2.6)	-0.1 (-0.3, 0.1)
Breast	0.6 (0.4, 0.8)	0.4 (0.2, 0.7)
Cervix uteri	0.9 (0.2, 1.6)	-0.5 (-1.0, -0.1)
Corpus uteri	1.0 (0.5, 1.5)	-0.7 (-0.8, -0.5)
Ovary and tubes	-1.5 (-2.1, -0.9)	-1.7 (-1.9, -1.5)
Prostate	1.5 (0.3, 2.8)	-0.9 (-1.3, -0.6)
Kidney	2.7 (2.1, 3.3)	1.2 (0.9, 1.5)
Melanoma of skin	2.0 (1.2, 2.8)	4.2 (3.8, 4.6)
Thyroid	3.8 (3.2, 4.3)	4.0 (3.5, 4.5)
Non Hodgkin lymphoma	-0.1 (-0.4, 0.2)	1.0 (0.7, 1.3)
Multiple myeloma	1.2 (0.3, 2.1)	1.1 (0.9, 1.3)
Leukaemia	0.3 (-0.2, 0.8)	0.2 (-0.1, 0.4)

Dark orange: EAPC ≥2.0% (significant)
Orange: EAPC 1.0-1.9% (significant)
Yellow: EAPC 0.1-0.9% (significant)
White: EAPC non-significant
Blue: EAPC<0% (significant)

Supplemental Table S4. Number (N) of cancer cases and incidence rates (IR) per 100,000 per cancer site for ages 20-49 and 50-84 years, men and women combined (except for sex-specific cancers, breast, ovary and tubes, cervix uteri, corpus uteri and prostate), and over 5-year calendar periods, 2003-2022 Nordic countries.

	**20-49**				**50-84**			
**Cancer sites**	**2003-2007**	**2008-2012**	**2013-2017**	**2018-2022**	**2003-2007**	**2008-2012**	**2013-2017**	**2018-2022**
	**N (IR)**	**N (IR)**	**N (IR)**	**N (IR)**	**N (IR)**	**N (IR)**	**N (IR)**	**N (IR)**
Lip, oral cavity and pharynx	1511 (3.0)	1595 (3.1)	1594 (3.1)	1512 (2.8)	10036 (24.3)	12273 (28.0)	14446 (30.8)	16454 (32.8)
Stomach	890 (1.8)	793 (1.6)	815 (1.6)	861 (1.6)	11331 (27.4)	10423 (23.8)	10580 (22.5)	11413 (22.7)
Colon	2360 (4.8)	2680 (5.3)	3188 (6.1)	3658 (6.9)	41158 (99.6)	45731 (104.4)	53370 (113.7)	56146 (111.8)
Rectum	1471 (3.0)	1608 (3.2)	1880 (3.6)	1958 (3.7)	23295 (56.4)	24308 (55.5)	27197 (58.0)	27165 (54.1)
Liver	353 (0.7)	407 (0.8)	411 (0.8)	471 (0.9)	5383 (13.0)	6879 (15.7)	8992 (19.2)	10516 (20.9)
Pancreas	733 (1.5)	743 (1.5)	767 (1.5)	848 (1.6)	14683 (35.5)	16299 (37.2)	19362 (41.3)	21776 (43.4)
Lung	2323 (4.7)	2098 (4.1)	1736 (3.3)	1508 (2.8)	56052 (135.7)	62435 (142.5)	68288 (145.5)	73213 (145.8)
Breast	15891 (65.2)	16518 (66.5)	17394 (68.1)	17532 (67.7)	64106 (296.7)	73494 (324.4)	78654 (326.8)	83258 (324.8)
Cervix uteri	3269 (13.4)	3574 (14.4)	4187 (16.4)	3821 (14.7)	2993 (13.9)	2692 (11.9)	2935 (12.2)	3171 (12.4)
Corpus uteri	920 (3.8)	976 (3.9)	1038 (4.1)	1076 (4.2)	15292 (70.8)	16184 (71.4)	16718 (69.5)	17171 (67.0)
Ovary and tubes	1594 (6.5)	1448 (5.8)	1322 (5.2)	1335 (5.2)	9533 (44.1)	9563 (42.2)	9451 (39.3)	8971 (35.0)
Prostate	855 (3.4)	1179 (4.6)	1321 (5.0)	1116 (4.1)	101025 (512.4)	109893 (519.6)	118775 (519.7)	121363 (493.8)
Kidney	1232 (2.5)	1590 (3.1)	1841 (3.5)	1882 (3.5)	12365 (29.9)	14551 (33.2)	17436 (37.2)	18804 (37.5)
Melanoma of skin	7306 (14.7)	9683 (19.1)	10940 (20.9)	10224 (19.2)	18564 (44.9)	26086 (59.5)	34160 (72.8)	43064 (85.8)
Thyroid	2279 (4.6)	2897 (5.7)	3862 (7.4)	4079 (7.7)	3075 (7.4)	3844 (8.8)	5350 (11.4)	6639 (13.2)
Non Hodgkin lymphoma	2447 (4.9)	2517 (5.0)	2548 (4.9)	2503 (4.7)	15906 (38.5)	18716 (42.7)	21688 (46.2)	23713 (47.2)
Multiple myeloma	390 (0.8)	475 (0.9)	490 (0.9)	478 (0.9)	6919 (16.7)	7916 (18.1)	9223 (19.7)	10618 (21.1)
Leukaemia	1667 (3.4)	1796 (3.5)	1885 (3.6)	1803 (3.4)	11371 (27.5)	12300 (28.1)	14240 (30.3)	14757 (29.4)
Total	47491 (95.7)	52577 (103.8)	57219 (109.5)	56665 (106.6)	423087 (1024.0)	473587 (1081.1)	530865 (1131.3)	568212 (1131.8)

Supplemental Figure S1. Time trends (2003-2012 and 2013-2022, separately) in age-specific EAPC for cancer of prostate and melanoma of skin.

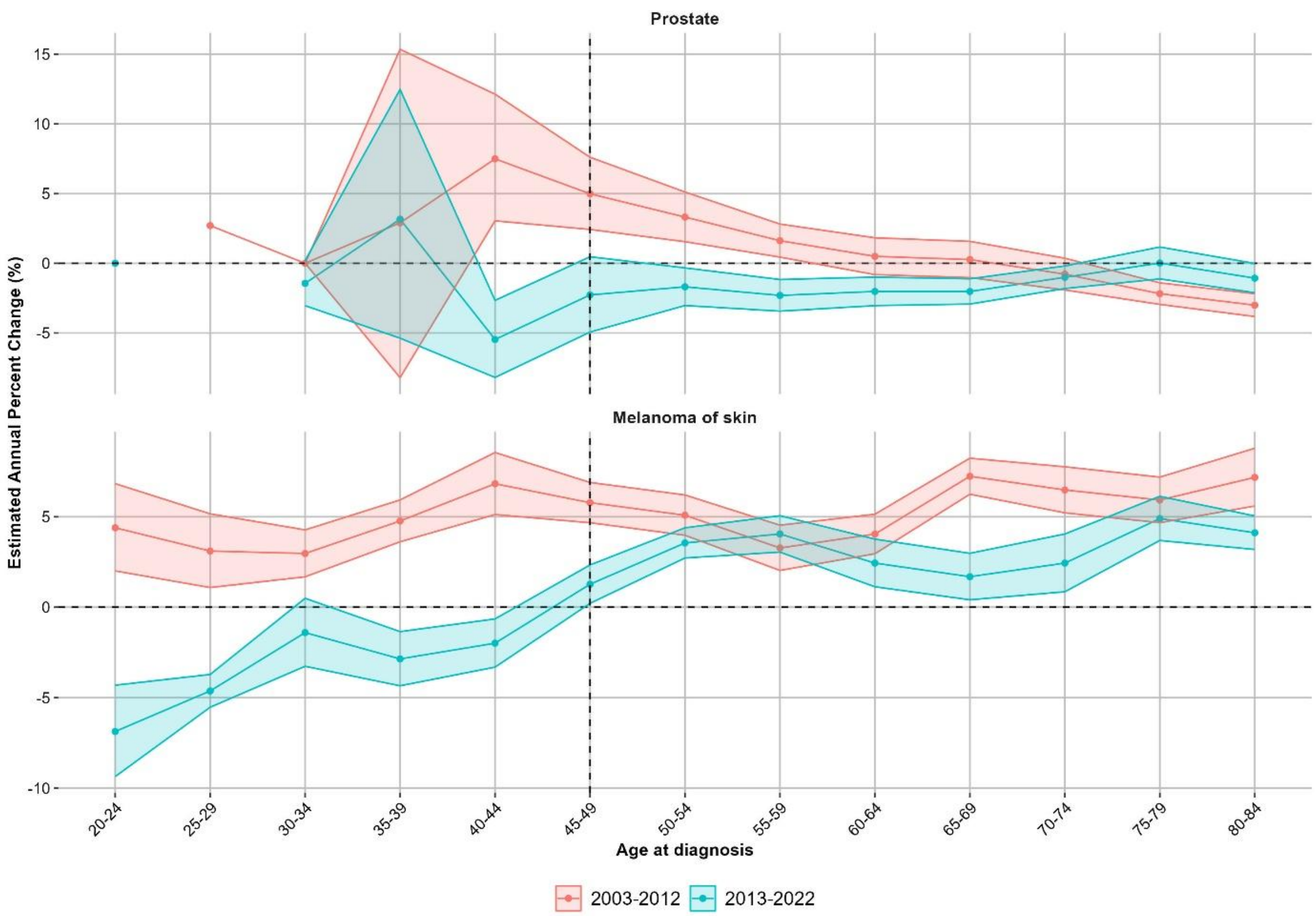

Supplemental Figure S2. Age-specific Estimated Annual Percent Change (%) per cancer site for **men**.

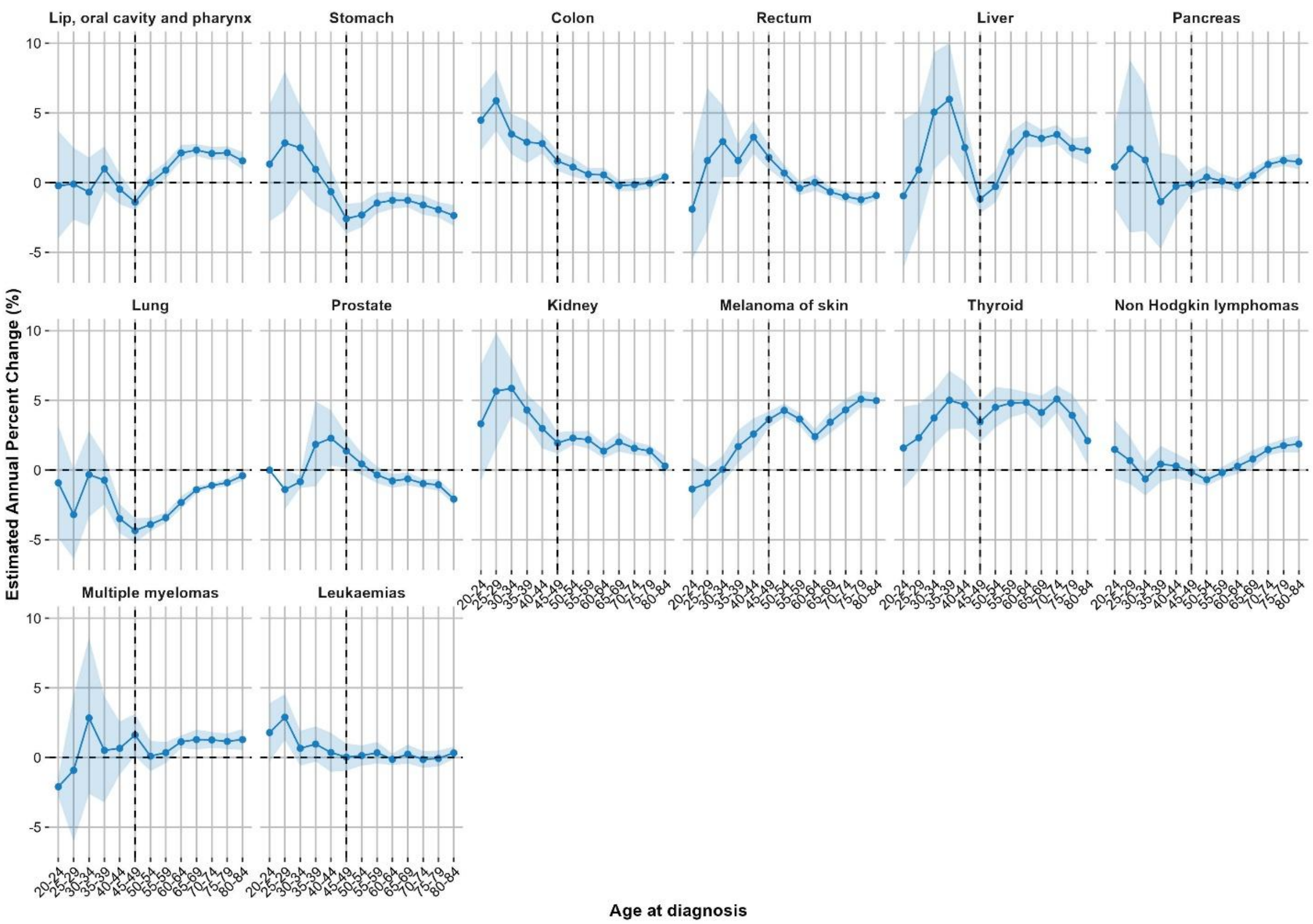

Supplemental Figure S3. Age-specific Estimated Annual Percent Change (%) per cancer site for **women**.

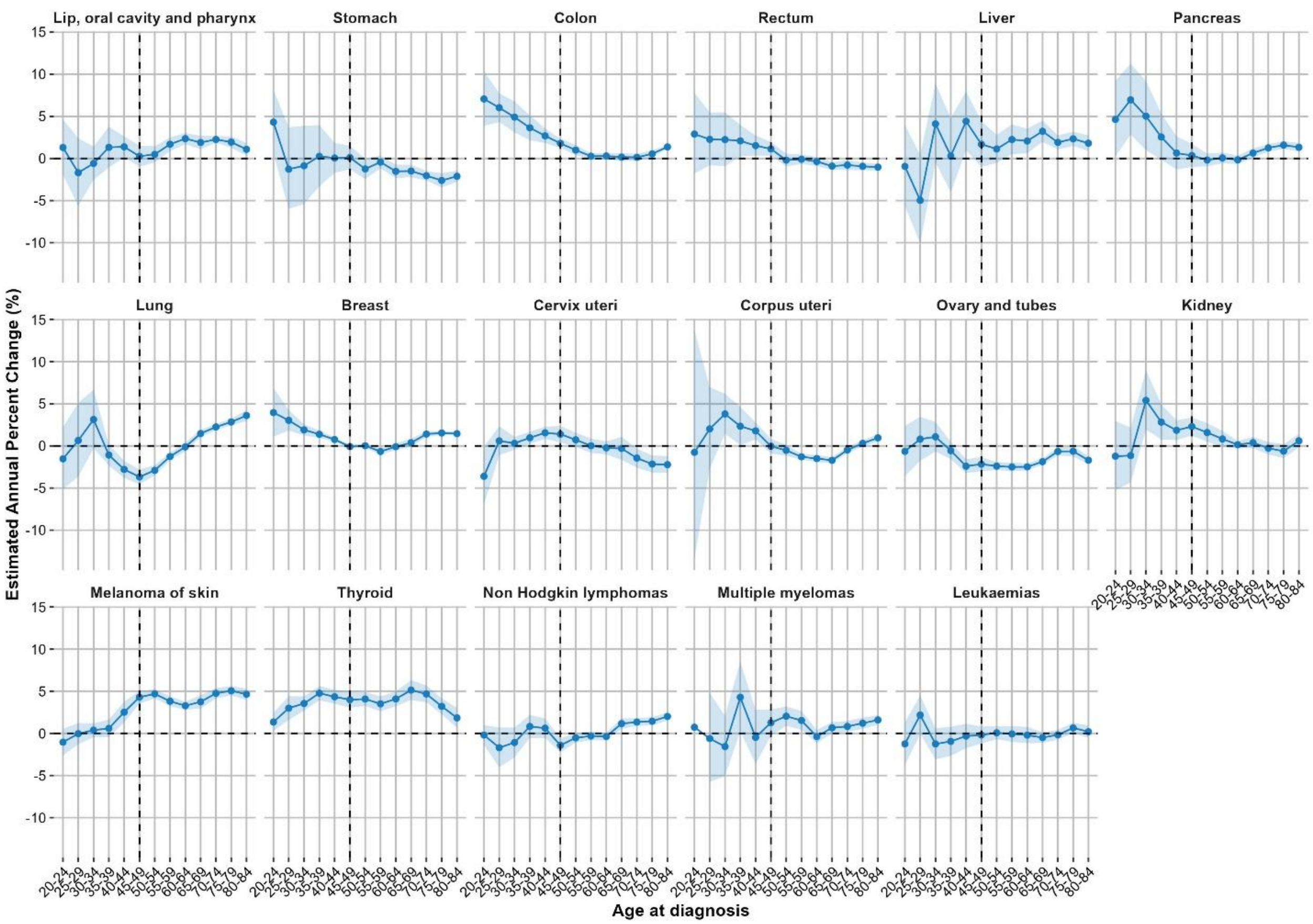

Figure S4. Age-specific incidence trends across period of diagnosis by cancer site, Nordic combined population for the nine sites with no or less pronounced increases in early-onset cancer incidence.

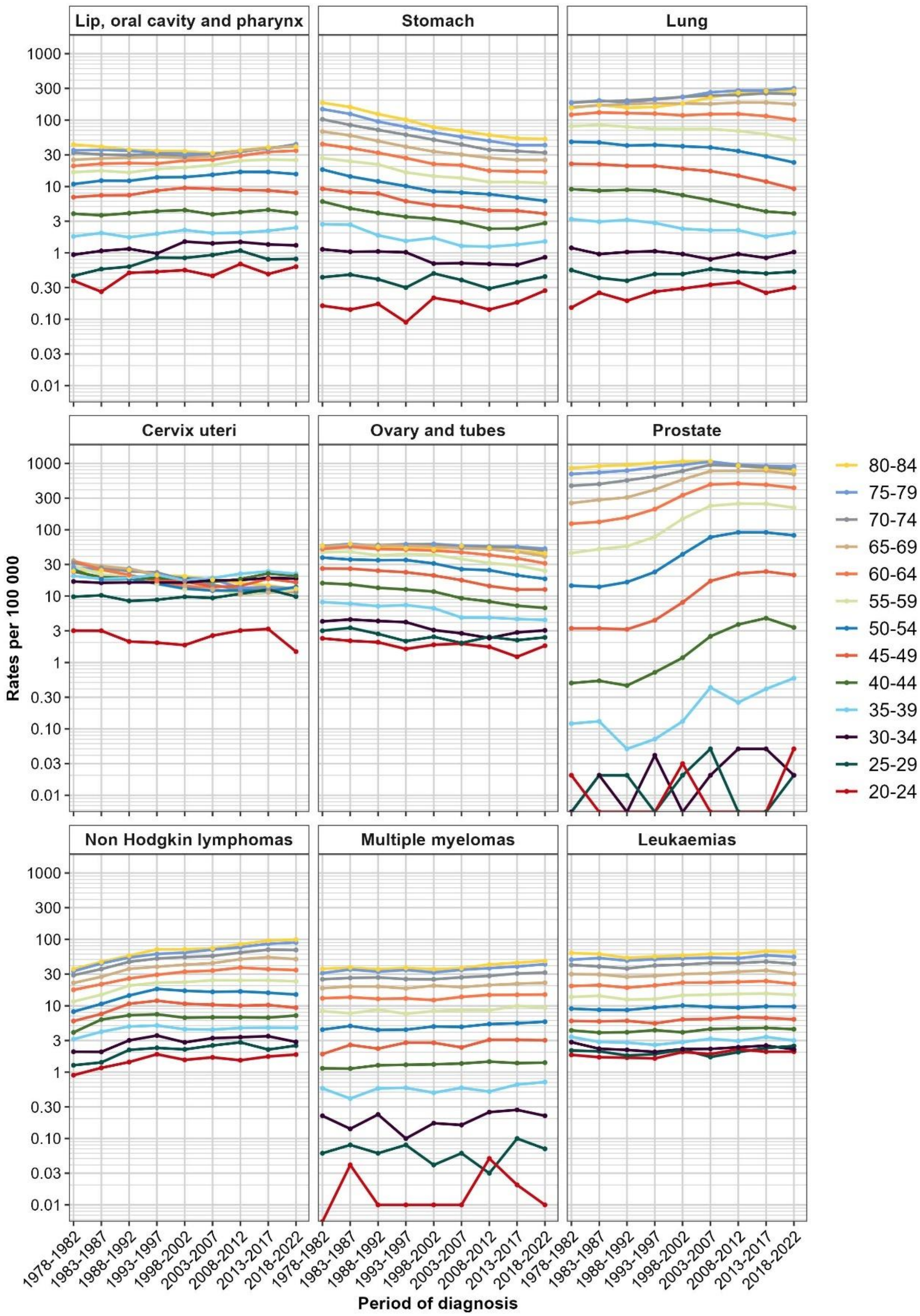

Figure S5. Age-specific incidence trends across birth cohort by cancer site, Nordic combined population for the nine sites with no or less pronounced increases in early-onset cancer incidence.

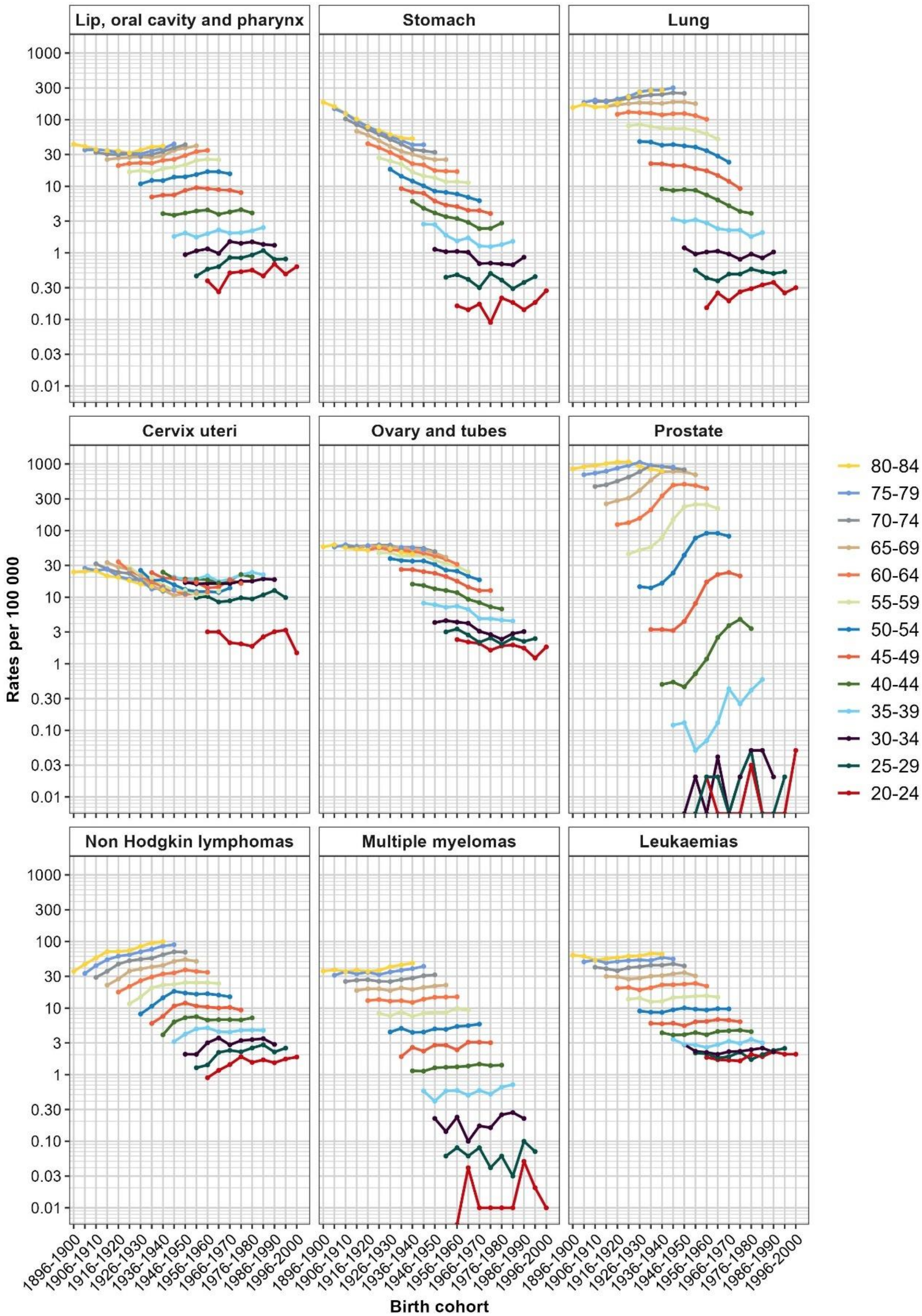

Supplement: Supplementary file 1 [file mmc1.pdf]
